# Alloxan-Induced Diabetes Triggers the Development of Periodontal Disease in Rats

**DOI:** 10.1371/journal.pone.0001320

**Published:** 2007-12-19

**Authors:** Marcela Claudino, Danielle Santi Ceolin, Sandra Alberti, Tania Mary Cestari, César Tadeu Spadella, Izabel Regina Fischer Rubira-Bullen, Gustavo Pompermaier Garlet, Gerson Francisco de Assis

**Affiliations:** 1 Department of Biological Sciences, School of Dentistry of Bauru, São Paulo University, Bauru, São Paulo, Brazil; 2 Department of Stomatology, School of Dentistry of Bauru, São Paulo University, Bauru, São Paulo, Brazil; 3 Department of Surgery and Orthopedics, School of Medicine of Botucatu, Sao Paulo State University, Botucatu, São Paulo, Brazil; Newcastle University, United Kingdom

## Abstract

**Background:**

Periodontal disease in diabetic patients presents higher severity and prevalence; and increased severity of ligature-induced periodontal disease has been verified in diabetic rats. However, in absence of aggressive stimuli such as ligatures, the influence of diabetes on rat periodontal tissues is incompletely explored. The aim of this study was to evaluate the establishment and progression of periodontal diseases in rats only with diabetes induction.

**Methodology/Principal Findings:**

Diabetes was induced in Wistar rats (n = 25) by intravenous administration of alloxan (42 mg/kg) and were analyzed at 1, 3, 6, 9 and 12 months after diabetes induction. The hemimandibles were removed and submitted to radiographical and histopathological procedures. A significant reduction was observed in height of bone crest in diabetic animals at 3, 6, 9 and 12 months, which was associated with increased numbers of osteoclasts and inflammatory cells. The histopathological analyses of diabetic rats also showed a reduction in density of collagen fibers, fibroblasts and blood vessels. Severe caries were also detected in the diabetic group.

**Conclusions/Significance:**

The results demonstrate that diabetes induction triggers, or even co-induces the onset of alterations which are typical of periodontal diseases even in the absence of aggressive factors such as ligatures. Therefore, diabetes induction renders a previously resistant host into a susceptible phenotype, and hence diabetes can be considered a very important risk factor to the development of periodontal disease.

## Introduction

Diabetes mellitus is a systemic disease characterized by abnormal metabolic regulation of both glucose and lipids, resulting in hyperglicemia and hyperlipidemia [1], [2]. In spite of some controversies, a positive correlation between diabetes and periodontal disease has been demonstrated [3], [4] with periodontal disease considered the sixth most common complication of diabetes mellitus [5]. Despite wide discussion in the literature regarding an association between diabetes and periodontal disease, the periodontal histological alterations induced by diabetes are poorly known [6], [7]. Diabetes seems to interfere in extracellular matrix metabolism in periodontium, reducing collagen synthesis [8] and increasing collagenolytical activity [9]. Histological studies also demonstrated increased vessel wall thickening in gingival tissue of diabetic patients [6], [10], [11]. In addition, some studies suggest diabetes seems to interfere in host defense mechanisms such as chemotaxis, adherence, phagocytosis and apoptosis, contributing therefore to tissue destruction [12], [13].

Although some authors have not found significant association between diabetes and periodontal disease in humans [14], [15] experimental models have demonstrated that the prevalence and severity of bone resorption in periodontal disease induced by ligatures or bacterial inoculation was higher in the presence of diabetes [16]–[18]. Experimental studies demonstrate that the increased severity of periodontal disease in diabetic animals was due to an exacerbated inflammatory response triggered by advanced glycation end products (AGEs) [16], [17], [19].

However, in all this previous experimental studies, the putative effect of diabetes over the periodontal tissues was always investigated in a scenario resultant of the deliberated induction of periodontal disease by means of bacterial inoculation or by silk ligatures [6], [7]. Nevertheless, recent studies suggest that diabetes could also favor, or even trigger, the establishment of periodontal disease, and not only exacerbate the established disease [20].

Therefore, considering that diabetes presents a great influence on periodontal health, and the putative diabetes effects over periodontium were never previously investigated without simultaneous intentional induction of periodontal disease, in this study we investigated the kinetics of radiographic and histological changes in periodontal tissues after diabetes induction in rats.

## Results

### Radiographic analysis

**Figure 1:**
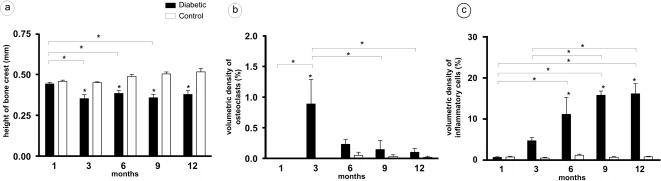
Increases of osteoclasts and inflammatory cells associated with alveolar bone loss in diabetic groups. Diabetic rats presented significant alveolar bone loss (a) starting at 3 months after alloxan injection when compared to the controls. After histotechnical procedures, sections were investigated by morphometric analyses for: b) volumetric density of osteoclasts, c) volumetric density of inflammatory cells.

Radiographic analysis was performed in order to evaluate alveolar bone levels throughout the course of experimental diabetes. Our data demonstrate that diabetic rats presented significant alveolar bone loss starting at 3 months after alloxan injection when compared to controls (Fig. 1). Similarly, alveolar bone levels were significantly lower in the diabetic group at 6, 9 and 12 months (Fig. 1). There were no statistically significant variations in the height of alveolar bone crest in the control group in any experimental period.

### Histological analysis

**Figure 2:**
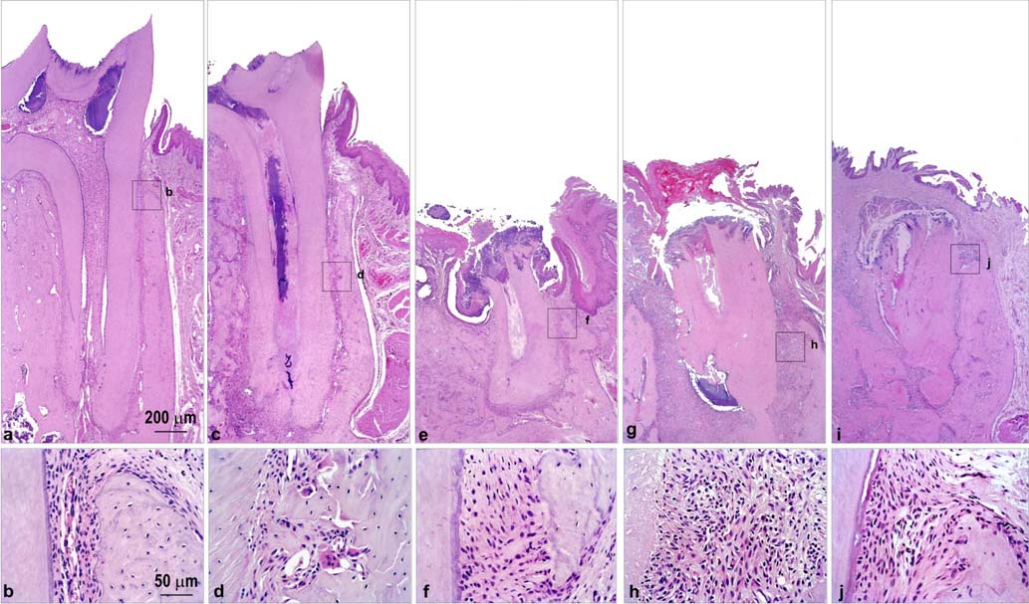
Diabetic rat presents typical histological alterations in the development of caries and periodontal disease. Diabetic rats were evaluated for dental and periodontal histological changes at 1 (a, b), 3 (c, d), 6 (e, f), 9 (g, h) and 12 (i, j) months after alloxan administration.

**Figure 3:**
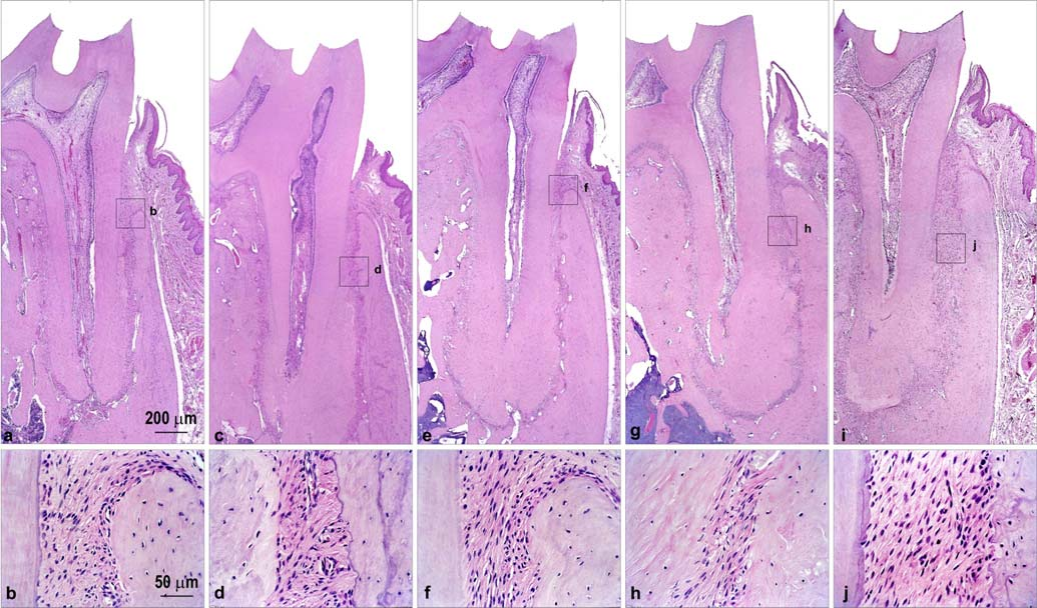
Absence of pathological changes in dental and periodontal tissues in control group. Animals were evaluated for dental and periodontal histological changes at 1 (a, b), 3 (c, d), 6 (e, f), 9 (g, h) and 12 (i, j) months after alloxan administration.

The experimental group showed evidence of development and progression of periodontal disease (Fig. 2), observed from the third month of diabetes induction. Histological evidence of dental caries was verified from the first month of follow-up in diabetic rats, increasing gradually during the disease course. At 6, 9 and 12 months all diabetic rats showed intense cariogenic activity. No evidence of dental caries was observed in control rats at any time analyzed (Fig. 3).

**Figure 4:**
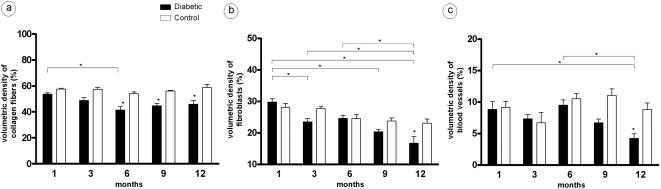
Reduction of volumetric density of collagen fibers, fibroblasts and blood vessels in diabetic groups. Histological sections of diabetic rats were investigated by morphometrical analyses for: a) volumetric density of collagen fibers, b) volumetric density of fibroblasts, c) volumetric density of blood vessels.

Compared to the control group, diabetic rats showed a significant reduction in volumetric density of collagen fibers at 6, 9 and 12 months (Fig. 4). Furthermore, in the latter period analyzed (12 months), fibroblasts and blood vessels also showed a volumetric density reduction (Fig. 4).

A significant but transitory increase in percentage of osteoclasts was detected at the third month, returning to initial levels after this time (Fig. 1), the same period when a significant increase in number of inflammatory cells in periodontium was verified. In addition, inflammatory infiltrate also showed increase in volumetric density at 6, 9 and 12 months in the diabetic group (Fig. 1).

Other components of conjunctive tissue, which includes space possibly occupied by inflammatory exudates, intercellular liquid and amorphous fundamental substance, did not present differences between diabetic and control groups.

Intragroup analyses did not show significant differences between measurements. The casual error was considered low, being 0.012 mm for height of bone crest evaluation, and casual error values ranged between 0 and 4.588% in histological analyses.

## Discussion

The relationship between diabetes and periodontal disease in humans is widely reported [21], [22]. It has been confirmed by experimental studies that diabetes increases the severity of periodontal disease induced by ligatures or bacterial inoculation [16]–[18]. However, the influence of diabetes on periodontium without external additional interferences and the potential histological changes throughout the course of diabetes are unknown.

Our results showed that diabetes induction, even without the intentional induction of periodontal disease by means of bacterial inoculation or by silk ligatures, results in alveolar bone loss, the main feature of periodontal disease development, beginning in the third month after diabetes induction. Previous studies have related absence of alveolar bone resorption in rats one month after diabetes induction, but longer periods were not evaluated [18], [23], [24]. Therefore, it is possible that longer periods of experimental diabetes may be necessary to trigger periodontal disease onset in the absence of inductive external agents of periodontal disease such as ligatures and bacterial inoculation. Another study showed that Goto-Kakizaki rats, a strain genetically prone to the development of type II diabetes, presented more bone resorption than Wistar rats even without the induction of periodontal disease [25]. However, since this study compared two different species of rats with different rates of physiologic bone tournover, its difficult to attribute the differential bone loss exclusively to be due the diabetes [25].

Our results showed that bone resorption occurred concomitantly with transitory increase in volumetric density of osteoclasts in a 3-month period. Current knowledge states that the migration of inflammatory cells expressing RANKL to periodontal tissues can trigger alveolar bone loss [26], [27]. Accordingly, our results also demonstrate that the volumetric density of inflammatory cells increased progressively from the 3^rd^ to 9^th^ month, stabilizing when reaching 12 months. In agreement with these authors, our results demonstrate that the presence of inflammatory cells in periodontium correlated positively with volumetric density of osteoclasts, and with alveolar bone loss values. In fact, the return to initial counts of inflammatory cells and osteoclasts is concomitant with the stabilization of bone resorptive process.

It is possible that persistence of inflammatory infiltrate is related to the presence of diabetes, since diabetes leads to intense and prolonged inflammatory responses, possibly due to accentuated inflammatory cytokine expression [28], [29]. Accordingly, diabetic patients present increased expression of cytokines such as IL-1b, IL-6 and TNF-α [30]–[32].

However, since periodontal disease onset takes place without the presence of ligatures or bacterial injection, it is possible that changes in the oral flora, or even in inflammatory immune response raised against oral bacteria may be involved in initiating the disease. It is also important to consider that AGEs accumulation in periodontal tissue could be involved in the development of inflammatory reaction and alveolar bone loss presented by the diabetic rats [16]. Another possible reason for the transitory bone resorptive activity in periodontium of diabetic mice may be the development of immunoregulatory mechanisms, which are thought to prevent or attenuate alveolar bone loss [33], [34].

Beyond this transitory alveolar bone loss period, our results showed significant long-standing changes in extracellular matrix and other cellular components of periodontium throughout the course of diabetes. A significant reduction in volumetric density of collagen fibers was found six months after diabetes induction, remaining stabilized until 12 months. The volumetric density of fibroblasts presented a significant reduction at the 12-month period in the diabetic group. According to our results, reduction of collagen fibers and fibroblasts was observed in gingival connective tissue of diabetic patients [10]. This reduction is supposed to result from diabetes-induced increased collagenase activity, and also by the increase in other enzymes related to degradation of extracellular matrix components [7]–[9].

In addition to enhanced enzymatic activity, the reduction of fibroblasts may also contribute to a reduced number of collagen fibers in the tissue of diabetic rats [10]. This reduction can be due to the capacity of diabetes to promote apoptosis of matrix-producing cells like osteoblasts and fibroblasts [35], [36]. Therefore, a significant reduction in tissue neoformation can be related to increased apoptosis of fibroblasts and results in impaired tissue repair [35].

By contributing to impairment of the repair process, diabetes is involved in the reduction of angiogenesis. In fact, our results showed reduction in volumetric density of blood vessels at 12 months. Accordingly, diabetes reduces the expression of angiogenic cytokines while increases the antiangiogenic mediators [37], [38]. Diabetes also interferes in angiogenesis by reducing the number of circulating endothelial progenitor cells, decreasing blood vessel neoformation and impairing the repair process [39].

In addition to periodontal disease, accentuated presence of caries was observed in all periods in the diabetic group showing progressive evolution from the 1st month. Some authors described, in the presence of diabetes, increased prevalence of caries and reduction in salivary flow rates [40]. In accordance, under similar conditions of oral hygiene and salivary flow, diabetic patients usually present higher incidence of caries than the control subjects [41]. Previous studies also demonstrated that poorly controlled diabetics patients present high prevalence of caries and low salivary flow rates [42]. Acordingly, diabetic rats present high incidence of caries one year after diabetes induction, but the progression of caries development throughout the course of diabetes was not evaluated [23]. We demonstrated that dental caries can be detected in early periods, starting at 3 months after diabetes induction, and a progressive increase in their severity was observed along disease development. These high incidence of caries in diabetic rats possibly occurs in response to qualitative and quantitative salivary alterations, and/or due to a persistent hyperglycemia that could affect quantitatively the microbiological oral profile of the rats [23], [40], [43], [44].

However, the mechanisms by which diabetes induced periodontal disease remain to be elucidated. It is possible that development of diabetes results in an accentuated inflammatory phenotype that, in response to normal flora in periodontal biofilm, initiates an inflammatory process that leads to periodontal destruction. Alternatively, diabetes-induces changes in the oral flora, or the accumulation of AGEs in gingival tissues may also be related with the development of periodontal disease by diabetic rats. Moreover, alterations in repair process resultant from diabetes, such as alterations in fibroblast biology and collagen synthesis, probably contribute to periodontal tissue destruction in diabetic mice.

The results presented here demonstrate that diabetes induction trigger alterations which are typical of periodontal diseases even in the absence of aggressive factors such as ligatures. Therefore, diabetes induction renders a previously resistant host into a susceptible phenotype, thus triggering the development of periodontal disease. Consequently, diabetes can be considered a very important risk factor to periodontal disease since it could also trigger, or even co-induce (as suggested by Golub et al., 2006 [20]) the onset of periodontal disease, and not only exacerbate the established disease. However, other studies must be carried out to improve knowledge on the interaction between diabetes and periodontal diseases, which may serve as a basis for development of more effective strategies for prevention and treatment of periodontitis in diabetic patients.

## Materials and Methods

### Induction of diabetes and collection of samples

Adult male Wistar rats, 10–12 weeks old, weighing approximately 250 g were rendered diabetic by intravenous administration of alloxan (Sigma Chemical Co., St Louis, EUA) in a single dose of 42 mg/kg of body weight into the caudal vein. Only rats showing two successive determinations of blood glucose levels (7 and 14 days after drug injection) greater than 250 mg/dL were considered diabetic and included in the experiment. The glucose levels during the experiment were typically 400–450 mg/dl in diabetic rats. Control rats were treated identically, except that saline solution was injected, and presented serum glucose levels that ranged from 90–130 mg/dL. The animals were housed in metabolic cages in groups of four per cage, and fed a standard rat chow and water *ad libitum*. Study protocol was approved by the Experimental Committee of Bauru School of Dentistry, São Paulo State University.

Each diabetic and control group was composed of 25 animals, subdivided into 5 subgroups, analyzed at 1, 3, 6, 9 and 12 months after diabetes induction. On their respective sacrifice dates, the animals were anaesthetized with 30 mg/kg of sodium pentobarbital (Cristália Chemical Products – Itapira/SP) and the hemimandibles were removed and fixed in 10% formalin solution for 7 days.

### Radiographic procedures

After fixation, the hemimandibles were radiographed with X-707 (Yoshida Dental MFC Co. Ltd., Tokyo, Japan) at 70 kvp, 7 mA with exposure time of 0.17 s and 40 cm source-to-film distance (exposure of the lingual surface of each hemimandible). The radiographs were digitized (laser scanner Lumiscan 50, Lumisys, Brazil) and evaluated by software Image J 1.38 (ImageJ, National Institutes of Health, Bethesda, Maryland, USA). The bone crest height was obtained by measuring a line traced perpendicular to the occlusal plane from the most coronal region of bone crest to the jaw base.

### Histological analyses

After the fixation and radiographical procedures, the hemimandibles were descalcified for 8 weeks in 18% EDTA., washed, dehydrated and embedded in paraffin. Serial sections (5 semi-serial sections of each hemimandible) with 5 µm thickness were cut and stained with haematoxilin and eosin. In the sequence, all histological sections were identified with a random numerical sequence in order to codify experimental periods and groups during the analysis procedures, and the 1^st^, 3^rd^ e 5^th^ were analyzed by a single calibrated investigator with a binocular microscope (Olympus Optical Co., Tokyo, Japan).

Morphometric measurements were obtained using a 100× immersion objective and a Zeiss kpl 8 X eyepiece containing a Zeiss II integration grid (Carl Zeiss Jena GmbH, Jena, Germany) with 10 parallel lines and 100 points in a quadrangular area. The grid image was successively superimposed on approximately 15 histological fields per histological section, comprising all the periodontal area, from the junctional epithelium to the root apex of mesial face of lower first molar mesial root. For each animal, 3 sections were counted, getting an average for each component.

In morphometric analyses, points were counted (Pi) coinciding with the images of the following components of periodontium (i): collagen fibers, fibroblasts, blood vessels, inflammatory cells, osteoclasts, bone, cement and other components of conjunctive tissue (inflammatory exudate, intercellular liquid and amorphous fundamental substance). Bone and cement scores were not considered since the region of interest was limited to the periodontium. Then, the total number of points (Pt) was obtained as well. Volume density was calculated by the equation: Vvi = Pi/Pt. Results were presented as the mean volume density for each structure considered in each examined group.

### Statistical analyses

A two-way ANOVA was employed to analyze radiagraphic and histological data from all groups, followed by one-way ANOVA and Tukey's test to determine significant differences within the same group related to time. The intragroup changes were analysed by paired t-test and the Dahlberg formula. The significance level was always set at p<0.05 and all calculations were performed using GraphPad program Prism 3.0 (GraphPad Software Inc, EUA).
